# An Accurate and Fault-Tolerant Target Positioning System for Buildings Using Laser Rangefinders and Low-Cost MEMS-Based MARG Sensors

**DOI:** 10.3390/s151027060

**Published:** 2015-10-23

**Authors:** Lin Zhao, Dongxue Guan, René Jr. Landry, Jianhua Cheng, Kostyantyn Sydorenko

**Affiliations:** 1 Marine Navigation Research Institute, College of Automation, Harbin Engineering University, Harbin 150001, China; E-Mails: zhaolin@hrbeu.edu.cn (L.Z.); ins_cheng@163.com (J.C.); 2 LASSENA, École de Technologie Supérieure, 1100 Notre-Dame Street West, Montreal, QC H3C 1K3, Canada; E-Mails: renejr.landry@etsmtl.ca (R.J.L.); kostyantyn.sydorenko.1@etsmtl.net (K.S.)

**Keywords:** target positioning, AHRS, laser-aided system, FKF, fault-tolerant

## Abstract

Target positioning systems based on MEMS gyros and laser rangefinders (LRs) have extensive prospects due to their advantages of low cost, small size and easy realization. The target positioning accuracy is mainly determined by the LR’s attitude derived by the gyros. However, the attitude error is large due to the inherent noises from isolated MEMS gyros. In this paper, both accelerometer/magnetometer and LR attitude aiding systems are introduced to aid MEMS gyros. A no-reset Federated Kalman Filter (FKF) is employed, which consists of two local Kalman Filters (KF) and a Master Filter (MF). The local KFs are designed by using the Direction Cosine Matrix (DCM)-based dynamic equations and the measurements from the two aiding systems. The KFs can estimate the attitude simultaneously to limit the attitude errors resulting from the gyros. Then, the MF fuses the redundant attitude estimates to yield globally optimal estimates. Simulation and experimental results demonstrate that the FKF-based system can improve the target positioning accuracy effectively and allow for good fault-tolerant capability.

## 1. Introduction

Target positioning is a technique to obtain a geodetic three dimensional (3D) position of a non-contact object or point. The position of a target on a building (indoor or outdoor) is essential for civil and military applications, such as indoor mapping, localization of a building structure and precision guidance. A novel target positioning system based on a laser rangefinder (LR) and Micro Electro Mechanical Systems (MEMS) gyros was proposed by Cheng *et al.* [1]. MEMS gyros are used to derive the attitude of LR, and the LR is applied to measure the distance between the observer and the target. In addition, the LR’s attitude error dominates the positioning error. However, low-cost MEMS gyros suffer from inherently large noises due to the current fabrication limitations of MEMS technologies. This results in time-growing LR attitude errors. Therefore, developing accurate MEMS-based attitude determination is a key criterion for achieving an accurate low-cost target positioning system.

To limit the unbounded attitude errors resulting from gyros, an attitude sensor or system is usually used to aid gyros. The Global Positioning System (GPS) is used to aid an Inertial Navigation System (INS) in [1]. The INS/GPS attitude determination systems are applied in many fields [2,3,4]. However, GPS signals are blocked when a target is positioned indoors or in urban canyons. In this paper, two attitude aiding systems are introduced for the attitude determination in GPS-Denied (GPSD) environments. The first one is an accelerometer/magnetometer aiding system, and the second one is a LR aiding system.

The accelerometer/magnetometer aiding system together with gyros constitutes a Magnetic, Angular Rate, and Gravity (MARG) sensors-based Attitude Heading Reference System (AHRS). MEMS accelerometer/magnetometers have advantages in terms of cost, size, weight, and power consumption. They can provide bounded-error attitudes by sensing Earth’s gravity and geomagnetic field. Consequently, this aiding system can be applied even in GPSD environments due to the ubiquitous presence of gravity and geomagnetic field on Earth [5]. Therefore, MARG sensors-based AHRS have been widely used for numerous applications, such as aircraft navigation [2], tracking for human body orientation [5], mobile applications [6], and pedestrian navigation [7].

The LR attitude aiding system presented in this paper consists of three LRs, which are installed to be parallel to each other. When the LRs can project beams on a wall, the attitude of the LR can be obtained by the change of the measurements of the LRs. Moreover, LRs possess the high measurement accuracy (e.g., ±1.5 mm per 80 m [8]), so the LR aiding system can provide accurate attitude determination for target positioning.

In AHRS, a Kalman Filter (KF) is commonly used to obtain attitude estimates by fusing the outputs from gyros and aiding systems. Most of the existing KF models are designed to provide the three attitude angles, namely the pitch, roll and yaw angles. However, in target positioning, the needed attitude information is three functions of two variables, which are the pitch and yaw angles. Besides, the functions are equal to three elements of the Direction Cosine Matrix (DCM). To obtain the direct attitude estimates, compact DCM-based KF models must be redesigned.

Two DCM-based KFs can be designed based on the accelerometer/magnetometer and LR aiding systems, respectively. However, both the aiding systems face robustness challenges in real scenarios. For instance, when the accelerometer/magnetometer aiding system is close to a ferrous or magnetic object, the external magnetic disturbances can make AHRS inaccurate [9]. Besides, the LR aiding system has large errors where there are not planar wall constructions. Also, the low reflectivity of wall surfaces can make the LR aiding system unusable [10]. Therefore, the single KF will have large errors when the corresponding aiding system fails in practical environments.

Employing two KFs based on the two aiding systems can improve the system robustness. The two KFs can provide attitude estimates simultaneously. Then, a Federated Kalman Filter (FKF) [11] can fuse the estimates from the two KFs to yield a globally optimal solution. This sensor redundancy and the FKF configuration can allow the whole system to be fault-tolerant. Even if one of the aiding systems has faults, the whole system can work effectively.

To implement an accurate and fault-tolerant target positioning system, this paper proposes a FKF-based system using MARG sensors and LRs. The objectives of this paper are: (1) to propose an LR attitude aiding system to enable accurate attitude measurements; (2) design DCM-based KF models for attitude estimation in the target positioning; and (3) develop a fault-tolerant FKF to improve the system robustness.

After the literature review in Section 2, Section 3 presents the structure of the proposed target positioning system. Section 4 details the positioning algorithm and the aiding systems. In Section 5, the FKF algorithms are designed. The simulation and experimental results are presented in Section 6. Finally, Section 7 presents our conclusions.

## 2. Literature Review

In the following, we will focus on the work regarding target tracking or positioning based on sensor fusion. Also, researches on topics involved in the proposed target positioning system will be discussed, namely MARG-based AHRS algorithms, laser aiding approaches and sensor fusion algorithms.

### 2.1. Target Tracking/Positioning Based on Sensor Fusion

Target tracking and positioning systems aim to search for a target and localize its position. Multiple sensors are employed to provide original motion and position measurements of targets. Then sensor fusion is conducted to obtain optimal position estimates. In [12,13], a Pyroelectric Infrared Radiation (PIR) and Radio Frequency (RF) localization system provide position information which is fused by an inference-based algorithm. The system modification is developed to successfully locate robots and people. In [14,15], Unmanned Aerial Vehicles (UAVs) are used to locate targets by a vision-based system with fixed downward-looking cameras. A square root sigma point filtering is used to fuse multiple observations and achieve precise positioning. In [16], Shi *et al.* fused a camera and inertial sensors by using a particle filter for long-term pedestrian tracking. In [17], Jing *et al.* addressed the problem of non-linear and non-Gaussian estimation for tracking a random moving object. An Improved Particle Filter (IPF) is designed to estimate the system motion parameters by processing the measurements from a sonar sensor and camera. In [18], laser scanners and a stereo vision camera are used to estimate the pose of moving objects in terrestrial and space applications. Based on the data from the Laser Camera System (LCS), [19] presents a closed-loop integrated sensor fusion approach, which consists of a Kalman filter and an Iterative Closest Point (ICP) algorithm. This method provides accurate and robust pose estimation in rendezvous and docking.

For the selection of sensors, Infrared Radiation (IR) sensors and RF localization system are available options for tracking multiple targets. However, IR signals are limited to the available line of sight. RF systems are imprecise in locating targets [12]. Vision sensors (e.g., cameras) can provide accurate target position information, but the corresponding camera modeling and image processing algorithms are complex and time-consuming. The laser scanner is a reliable device to provide range and attitude information, but it is more expensive and larger than LRs. In our system, MARG sensors and LRs are selected. They have advantages such as low cost, small size, system simplicity and being infrastructure free. Meanwhile, the accelerometer/magnetometer aiding system can be widely applied, for example in GPSD environments. Also, LRs aiding systems can provide accurate attitude information due to their high measuring accuracy.

From the perspective of fusion algorithms, non-linear Bayesian estimators, for instance Extended KF (EKF) and Particle Filter (PF), can solve the problem of non-linear estimation in practical target tracking or positioning missions. These fusion algorithms eliminate the errors from a stand-alone sensor itself to improve accuracy. However, these non-linear Bayesian estimators suffer from computational complexity and robustness problems. In this paper, we design a fault-tolerant FKF-based fusion algorithm. The FKF consists of two local linear KFs and a master filter. Two linear KFs adopt the same DCM-based dynamic model to avoid nonlinear filtering. Meanwhile, two attitude aiding systems provide measurements for the two KFs, respectively. Each local KF can independently offer a rough attitude estimate. Then, the master filter processes this redundant attitude information to output a globally optimal attitude estimate. As a result, the aiding systems and FKF configuration confer the system fault-tolerant capability.

### 2.2. MARG-Based AHRS Algorithms

In our system, the MARG-based AHRS adopts gyros to sense the angular rates of the LR, which are required for solving the attitude differential equations [20]. Thus, the attitude derived from the gyros’ measurements displays time-growing error properties due to gyro noises. To overcome this problem, accelerometers and magnetometers are introduced to constitute an aiding system, which assist MEMS gyros in obtaining an accurate LR attitude via a KF estimator.

Previous researches have focused on the design of flexible Kalman filtering algorithms, which depend on different attitude descriptions. Based on Euler angle description, Emura and Tachi used a KF algorithm to track human head motions [21]. However, the Euler angles-based dynamic model suffers from singularity and nonlinearity. Quaternion-based KF [2] avoids singularity, however, nonlinearity still exists. In order to develop a linear KF that requires less computation than nonlinear KF, Han and Wang [22] proposed a KF algorithm based on Psi-angle error equations under a small-attitude-error assumption [23]. Meanwhile, a similar method is presented by Li and Wang in [24] with modification of the adaptive ability of the filter. However, the Psi-angle equations will be inaccurate when the attitude error accumulates to violate the small-attitude-error assumption, particularly when MEMS gyros operate continuously for a long time.

The elements of the DCM from the body frame to the local frame can be the KF states. The DCM-based KF can directly estimate the three elements of DCM, which are needed in our target positioning as illustrated in Section 4. In [25], DCM-based attitude estimation is presented to obtain the pitch, roll and yaw attitude angles, and it has successfully applied in land vehicle applications [26]. Two cascaded KFs were adopted to reduce the number of the KF states [25,26]. The DCM-based KF models are linear and free of singularity. In our target positioning system, the LR has no roll angles, so the DCM can be further simplified. We redesign the DCM-based KF models without the cascaded structure to achieve economical computation and precise attitude estimation for target positioning.

### 2.3. Laser Aiding Approaches

LRs can output accurate distance measurements. They can constitute a reliable independent attitude aiding system to MEMS gyros. However, LRs are only able to measure the range from an observer to a target. In order to realize LR-aided AHRS system, the key is to build the relationship between range measurements and the LR’s attitude.

Cheng *et al.* have presented a laser-aided attitude calibration method [1]. The 3D target position can be calculated by the range measurements and the attitude of the LR. The position error can be obtained when positioning an accurately known position. The attitude errors dominate the positioning error, thus the least squares method performs an estimation of attitude errors for calibration. However, limited knowledge of the target position restrains the application of this method. Besides, laser scanners can provide the changes of heading angle directly to aid autonomous relative navigation [27], and similar applications occur in indoor navigation [10]. Although a laser scanner allows the convenience of having heading-angle observations, it has high cost and large size compared to a LR.

In [28], two parallel laser pointers project points onto a plane surface. Then, the difference of the two distance measurements, the distance of the two lasers and the line on the plane surface form a right triangle. The laser distance measurements are used to compute the change of yaw angle of underwater vehicles. Inspired by the laser aiding method described in [28], we employ three LRs to compute the changes of pitch and yaw angles. Furthermore, combined with the initial attitude alignment, the pitch and yaw angles of the LR can be obtained.

### 2.4. Sensor Fusion Algorithms

Sensor fusion algorithms based on linear and nonlinear filters are widely applied in navigation and localization. The KF algorithm, as a linear estimator, is used to fuse the data from gyros, accelerometers and magnetometers for pedestrian navigation [29]. Non-linear EKF [30,31] and PF [32] are applied in indoor positioning and autonomous navigation. In [10,33], two cascaded EKFs are employed to process the different device noises and then improve the final accuracy. However, the whole system will be inaccurate or even invalid if any fault occurs in each filter. In our target positioning system, two linear KFs can be designed based on the accelerometer/magnetometer and LR aiding systems, respectively. Both KFs are capable of providing the attitude estimates. Then, a FKF can allow the whole system to be fault-tolerant by processing the redundant estimates.

Since FKF was proposed by Carlson in 1980s [11], it has been used in integrated navigation and target tracking [34] due to its efficiency and flexibility. A no-reset FKF configuration [35] is adopted in this paper to tolerate the faults of a single KF in practice, for instance when magnetic disturbances occur. In the no-reset FKF, there are no feedbacks from the master filter to reset the local filters. This no-reset configuration is discussed in [36], which is used to fuse the data of multiple inertial measurement units for pedestrian navigation.

The contributions of this paper are as follows: first, both accelerometer/magnetometer sensors and LRs are used to aid the MEMS gyros. The three LRs-based attitude aiding system is especially designed to provide precise attitude measurements. Second, the linear DCM-based KF models are modified for target positioning, which can avoid complex calculations. Meanwhile, the positioning accuracy is increased by the designed attitude estimation. Third, the no-reset FKF is adopted to process the redundant information of local filters. The FKF allows good system robustness. The system can output accurate target positions even if one of the aiding systems fails to work.

## 3. Overall Design of the Target Positioning System

**Figure 1:**
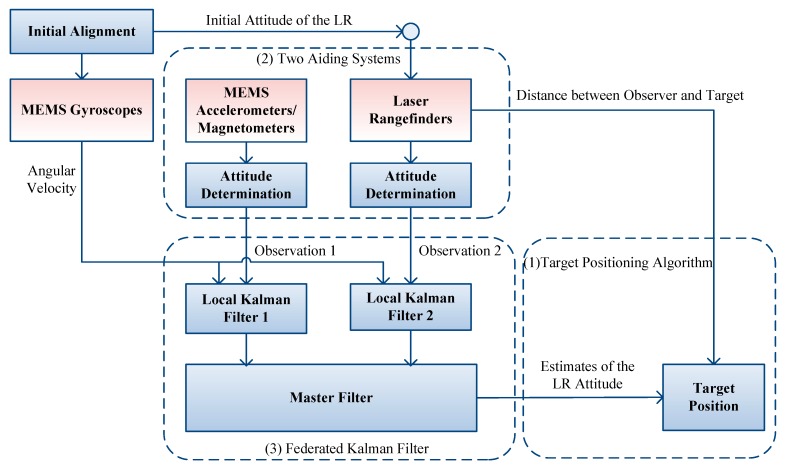
Structure of the proposed target positioning system.

The proposed target positioning system consists of MEMS gyros; MEMS accelerometers and magnetometers; and three LRs. To aid the MEMS gyros, attitude aiding systems based on accelerometer/magnetometer sensors and three LRs are designed, respectively. Besides constituting the LR aiding system, one of the three LRs is selected to measure the distance between an observer and a target. MEMS sensors are mounted on the LR to sense its angular and linear motion, and the magnetic field of its position. Figure 1 shows the structure of the target positioning system.

The involved algorithms include three parts: target positioning algorithm, two aiding systems for attitude determination, and FKF for data fusion. The basic functions of the main parts are:

(1) Target positioning algorithm: One of the three LRs is selected to provide the distance between the observer and the target. In our system, the target position can be obtained by the distance measurement from this LR and the attitude angles of this LR. The LR’s attitude angles are estimated by the FKF.

(2) Aiding systems for attitude determination: Both accelerometer/magnetometer and LR aiding systems can offer the LR’s attitude. These two aiding systems provide the observations for the two local KF 1 and 2 of the FKF, respectively.

(3) FKF for data fusion: MEMS gyros sense the angular rate of the LR. The angular rate is used to describe the dynamic models of the two local KFs 1 and 2. Combined with the observation models corresponding to the two aiding systems, the two local KFs can estimate the LR’s attitude simultaneously. Then, the master filter fuses the redundant estimates to output globally optimal estimates.

## 4. Target Positioning Algorithm and Aiding Systems

### 4.1. Target Positioning Algorithm

**Figure 2:**
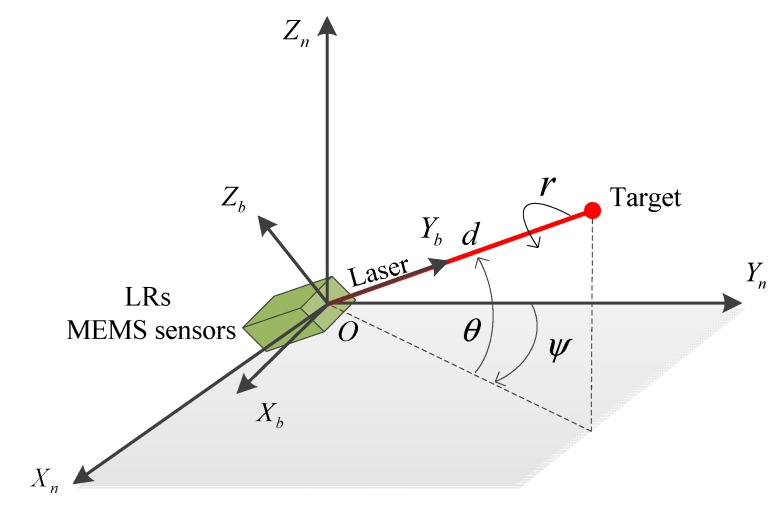
Target positioning using the proposed system.

The underlying idea of target positioning is presented as follows. First, the relation between a target and an observer in a frame is built using two major parameters, namely the distance between them and the line-of-sight angles (*i.e.*, azimuth and elevation angles) [37]. Second, provided the observer position within the frame, the target position can be computed with the built relation. Third, if required, the target position in an arbitrary frame can be obtained when knowing the transformation from the original frame to this arbitrary frame. The principle of the target positioning is shown in Figure 2.

The following frames are used in this paper [38]:

(1) The Earth frame (e-frame *OX_e_Y_e_Z_e_*): the frame origin is the Earth center and the axes *OX_e_*, *OY_e_*, and *OZ_e_* are fixed with respect to the Earth. The axis *OZ_e_* lies along the Earth’s polar axis, and the axis *OX_e_* lies along the intersection of the plane of the Greenwich meridian with the Earth’s equatorial plane. 

(2) The navigation frame (n-frame *OX_n_Y_n_Z_n_*): this frame is a local geographic frame, whose origin *O* is set at the LR location, and its axes are aligned with the directions of the North, East and the local vertical (up).

(3) The body frame (b-frame *OX_b_Y_b_Z_b_*)): this frame is an orthogonal axis set, whose origin is the LR mass center, and its axes are aligned with the roll, pitch and yaw axes of the LR.

An observer holds the target positioning system consisting of LRs and MEMS sensors. When positioning a target, the observer can rotate the system to make the LR point to the target and measure the distance *d* between the observer and the target. Meanwhile, MEMS sensors and LRs can provide measurements to calculate the LR’s attitude, which are the Euler angles describing the orientation of the b-frame relative to the n-frame. The Euler angles are denoted as pitch angle θ, roll angle *r* and yaw angle ψ. Besides, the laser beam is aligned with the *Y_b_* axis of b-frame. Then, the target position within the n-frame $p_{\text{n}}$ can be expressed as:

(1) $$p_{\text{n}}=C_{\text{b}}^{\text{n}}\cdot p_{\text{b}}$$

where $p_{\text{b}}=(0,d,0)$ is the target position in b-frame, and $C_{\text{b}}^{\text{n}}$ is the DCM from the b-frame to the n-frame, which is given by:

$$C_{\text{b}}^{\text{n}}=\left[\begin{array}{ccc} \cos r\cos\text{ψ}+\sin r\sin\text{ψ}\sin\text{θ} & \sin\text{ψ}\cos\text{θ} & \sin r\cos\text{ψ}-\cos r\sin\text{ψ}\sin\text{θ}\\ -\cos r\sin\text{ψ}+\sin r\cos\text{ψ}\sin\text{θ} & \cos\text{ψ}\cos\text{θ} & -\sin r\sin\text{ψ}-\cos r\cos\text{ψ}\sin\text{θ}\\ -\sin r\cos\text{θ} & \sin\text{θ} & \cos r\cos\text{θ} \end{array}\right]$$

By substituting $p_{\text{b}}$ and $C_{\text{b}}^{\text{n}}$ into Equation (1), the target position in the n-frame is given by the following expression:

(2) $$p_{\text{n}}={\left[\begin{array}{ccc} x & y & z \end{array}\right]}^{\text{T}}={\left[\begin{array}{ccc} d\sin\text{ψ}\cos\text{θ} & d\cos\text{ψ}\cos\text{θ} & d\sin\text{θ} \end{array}\right]}^{\text{T}}$$

When knowing the position of the observer within the n-frame, *i.e.*, the latitude φ and longitude λ, the DCM from n-frame to e-frame equals to:

(3) $$C_{\text{n}}^{\text{e}}=\left[\begin{array}{ccc} -\sin\text{λ} & -\sin\text{φ}\cos\text{λ} & \cos\text{φ}\cos\text{λ}\\ \cos\text{λ} & -\sin\text{φ}\sin\text{λ} & \cos\text{φ}\sin\text{λ}\\ 0 & \cos\text{φ} & \sin\text{φ} \end{array}\right]$$

Then, the target position vector with regard to e-frame $p_{\text{e}}$ can be calculated by:

(4) $$p_{\text{e}}=C_{\text{n}}^{\text{e}}\cdot p_{\text{n}}$$

Transformation from $p_{\text{n}}$ to $p_{\text{e}}$ can be considered as a separate topic from target positioning. Obtaining the LR position (*i.e.*, latitude φ and longitude λ) belongs to self-localization, which can be realized by mapping, GPS, INS, or GPS/INS solutions. Since self-localization is a separate topic from target positioning, this paper only focuses on improving the accuracy of $p_{\text{n}}$ in Equation (2).

Equation (2) implies the requirements for attitude in target positioning system. First, only the pitch angle θ and the yaw angle ψ perform the computation of the target position. The roll angle is generated by a rotation around laser beam (*Y_b_* axis of b-frame). The rotation around *Y_b_* causes no changes of the target position, so the LR only needs to rotate around *X_b_* and *Z_b_* axes of the b-frame to point toward the target. Second, instead of attitude, the functions of the attitude angles (*i.e.*, sinψcosθ, cosψcosθ and sinθ) are the direct factors influencing the target positioning. Besides, they are the elements in the second column of DCM $C_{\text{b}}^{\text{n}}$.

In [1], Cheng *et al.* has proved that the accuracy of $p_{\text{n}}$ is mainly affected by the errors of the LR attitude. MEMS gyros could sense the angular rates of each axis of the b-frame. By the integral of the gyros measurements, we can get the rough LR attitude. However, the MEMS gyros noises decrease the attitude accuracy, especially in a long-term operation. In MEMS-based AHRS, aiding systems are usually needed for offering bounded-error measurements. Then, the unbounded attitude errors from MEMS gyros can be limited via a KF, which blends the gyros measurements and aiding systems.

### 4.2. Aiding Systems for Attitude Determination

#### 4.2.1. Accelerometer/Magnetometer Aiding System

Accelerometers and magnetometers can be integrated as an attitude aiding system by sensing the gravity and the geomagnetic field, respectively. The LR stays stationary when pointing to a target. In this case, only the gravity $g_{\text{n}}$ acts on the LR, so the measurements from accelerometers *A_x_*, *A_y_* and *A_z_* represent the components of gravity along the axes of b-frame. The vector of gravity in b-frame $g_{\text{b}}$ can be expressed as:

(5) $$g_{\text{b}}=C_{\text{n}}^{\text{b}}g_{\text{n}}={\left(C_{\text{b}}^{\text{n}}\right)}^{\text{T}}\left[\begin{array}{c} 0\\ 0\\ -g \end{array}\right]=\left[\begin{array}{c} g\sin r\cos\text{θ}\\ -g\sin\text{θ}\\ -g\cos r\cos\text{θ} \end{array}\right]=\left[\begin{array}{c} A_{x}\\ A_{y}\\ A_{z} \end{array}\right]$$

Then, the roll angle *r* and pitch angle θ can be given by the accelerometer measurements:

(6) {θ=arcsin(−Ayg)r=arctan(−AxAz)

**Figure 3:**
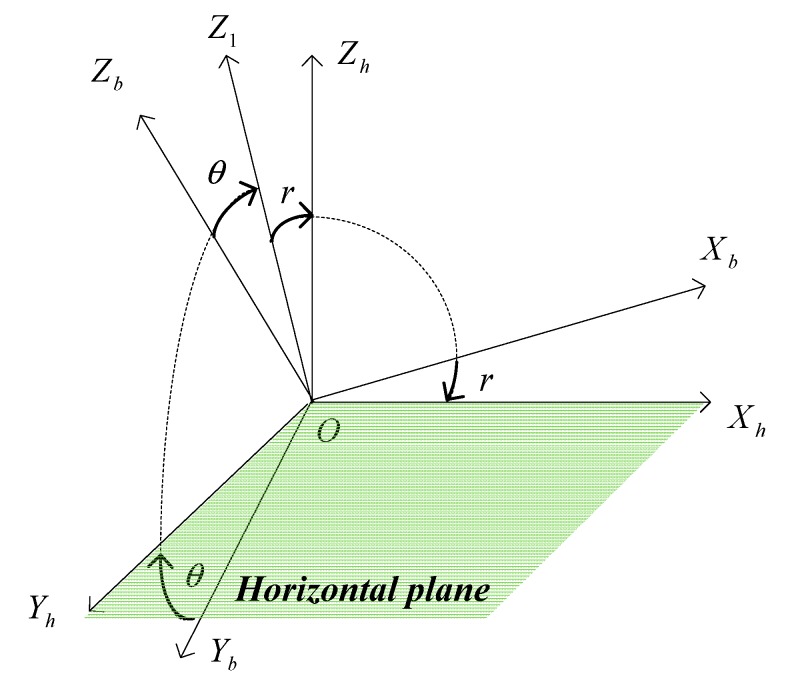
Transformation from the b-frame to the h-frame.

To compute the yaw angle ψ by using the measurements of geomagnetic field, a new frame h-frame is introduced. The h-frame is transformed from the b-frame by two rotations. The first rotation acts around *X_b_* axis with a pitch angle θ, which generates the coordinate *OX_b_Y_h_Z_1_*. The second rotation performs around *Y_h_* with the roll angle *r*, which generates the coordinate *OX_h_Y_h_Z_h_*. With this transformation, the h-frame has a horizontal plane *OX_h_Y_h_* as shown in Figure 3.

The Earth’s magnetic field vector in the b-frame ${\left[\begin{array}{ccc} M_{x}^{b} & M_{y}^{b} & M_{z}^{b} \end{array}\right]}^{\text{T}}$ are given by the magnetometer sensors. The Earth’s magnetic field vector in the h-frame ${\left[\begin{array}{ccc} M_{x}^{h} & M_{y}^{h} & M_{z}^{h} \end{array}\right]}^{\text{T}}$ can be calculated by:

(7) $$\left[\begin{array}{c} M_{x}^{h}\\ M_{y}^{h}\\ M_{z}^{h} \end{array}\right]={\left(\left[\begin{array}{ccc} \cos r & 0 & -\sin r\\ 0 & 1 & 0\\ \sin r & 0 & \cos r \end{array}\right]\cdot \left[\begin{array}{ccc} 1 & 0 & 0\\ 0 & \cos\text{θ} & \sin\text{θ}\\ 0 & -\sin\text{θ} & \cos\text{θ} \end{array}\right]\right)}^{\text{T}}\cdot \left[\begin{array}{c} M_{x}^{b}\\ M_{y}^{b}\\ M_{z}^{b} \end{array}\right]$$

The local Earth’s magnetic field has a fixed component on the horizontal plane pointing to the Earth’s magnetic North. The yaw angle ψ of LR can be defined as:

(8) ψ=arctan(MyhMxh)+D

where *D* represents the declination angle relative to the geographic North, which varies and depends on the given LR (observer) location [39].

As mentioned in Section 4.1, the LR only has the pitch angle θ and the yaw angle ψ in target positioning, so the attitude formulations can be simplified. Using Equations (6)–(8), the simplified attitude determination can be rewritten as:

(9) $$\{\begin{array}{c} \text{θ}=\arcsin\left(\frac{-A_{y}}{g}\right)\\ \text{ψ}=\arctan\left(\frac{M_{x}^{b}}{M_{y}^{b}\cos\text{θ}-M_{z}^{b}\sin\text{θ}}\right)+D \end{array}$$

Under stationary conditions, the accelerometer/magnetometer aiding system can provide the bounded-error attitude measurements, which can be used as observations for attitude estimation. Besides, the accelerometer/magnetometer aiding system can work even in a GPSD environment. Therefore, the accelerometer/magnetometer aiding system is selected for our target positioning.

However, when this aiding system suffers from external magnetic disturbances from magnetic or metallic materials, the yaw angle calculated by Equation (9) will contain errors. Instead of the magnetic disturbances detection [9], modeling and estimation [39,40], a LR attitude aiding system is introduced as second aiding system to solve this problem. This redundant LR aiding system has high accuracy, which enables the system to maintain precise during the magnetic disturbances.

#### 4.2.2. Laser Rangefinder Aiding System

A LR is originally used for measuring distance without direct relations with the attitude. To use LRs for attitude determination, the distance measurements need to contribute to describing the attitude. Inspired by [28], this paper proposes a LR attitude aiding for target positioning. The approach is different from the method in [28] in two aspects. First, the attitude angles here represent Euler angles from the b-frame to the n-frame. Second, the LR aiding system can determine both pitch and yaw attitude angles.

**Figure 4:**
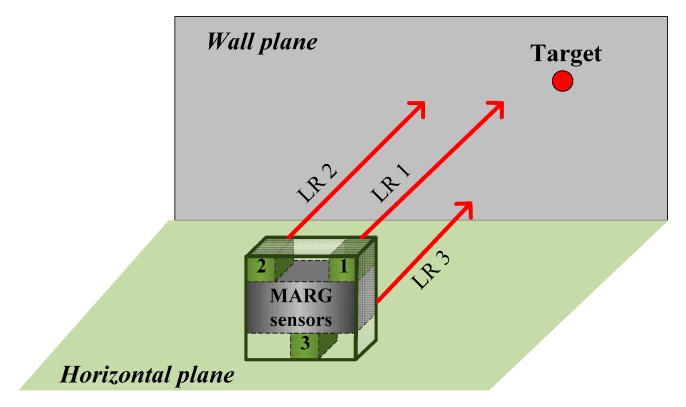
LR aiding system for attitude determination.

The LR aiding system consists of three identical LRs, which are installed in a box which encapsulates MEMS MARG sensors, as shown in Figure 4. The three LRs are installed to be parallel (aligned) to each other. LR 1 is selected to aim at the arbitrary target and provide the distance between the target and the observer. The measurements from the three LRs can be used for determining the attitude of LR 1 directly.

**Figure 5:**
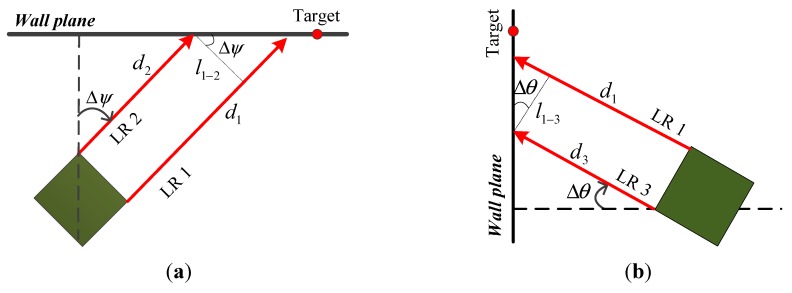
Calculation of the attitude changes. (**a**) Calculation of the yaw angle change; (**b**) Calculation of the pitch angle change.

Buildings commonly have two consistent construction features: (1) the floors are horizontal; (2) the walls are flat and vertical to the floors. Combined with these conditions, the distance measurements can structure right triangles, which are used for calculating attitude. Figure 5a,b explains how to obtain the changes of the yaw and pitch angles, respectively.

From Figure 5, the change of yaw angle Δψ and the change of pitch angle Δθ can be calculated by:

(10) $$\Delta \text{ψ}=\arctan\left(\frac{d_{1}-d_{2}}{l_{1-2}}\right),\;\;\;\Delta \text{θ}=\arctan\left(\frac{d_{1}-d_{3}}{l_{1-3}}\right)$$

where $d_{1}$, $d_{2}$ and $d_{3}$ are the measurements from LR 1, 2, and 3, respectively; $l_{1-2}$ represents the distance between LR 1 and 2; and $l_{1-3}$ represents the distance between LR 1 and 3. To ensure the calculated angles with good precision in practical use, the $l_{1-2}$ and $l_{1-3}$ should be determined by considering the measuring errors of LRs.

Considering the measuring errors of LRs, the change of yaw angle $\Delta \tilde{\psi }$ and pitch angle $\Delta \tilde{\theta }$ can be rewritten as:

(11) $$\begin{array}{l} \Delta \tilde{\psi }=\arctan\left(\frac{\left(d_{1}+\text{δ}d_{1})-(d_{2}+\text{δ}d_{2}\right)}{l_{1-2}}\right)=\arctan\left(\frac{\left(d_{1}-d_{2})+(\text{δ}d_{1}-\text{δ}d_{2}\right)}{l_{1-2}}\right)=\arctan\left(\frac{d_{1-2}+\text{δ}d_{1-2}}{l_{1-2}}\right)\\ \Delta \tilde{\theta }=\arctan\left(\frac{\left(d_{1}+\text{δ}d_{1})-(d_{3}+\text{δ}d_{3}\right)}{l_{1-3}}\right)=\arctan\left(\frac{\left(d_{1}-d_{3})+(\text{δ}d_{1}-\text{δ}d_{3}\right)}{l_{1-3}}\right)=\arctan\left(\frac{d_{1-3}+\text{δ}d_{1-3}}{l_{1-3}}\right) \end{array}$$

where $\text{δ}d_{1}$, $\text{δ}d_{2}$ and $\text{δ}d_{3}$ represent the measuring errors of LR 1, 2, and 3, respectively; $\text{δ}d_{1-2}$ and $\text{δ}d_{1-3}$ are the total measuring errors in $\Delta \tilde{\psi }$ and $\Delta \tilde{\theta }$, respectively; $d_{1-2}$ is the true difference between the measurements from LR 1 and 2; and $d_{1-3}$ is the true difference between the measurements from LR 1 and 3.

Equation (11) shows that the measuring errors $\text{δ}d_{1-2}$ and $\text{δ}d_{1-3}$ play the same role in $\Delta \tilde{\psi }$ and $\Delta \tilde{\theta }$, respectively. Hence, we analyze the Δψ error caused by the measuring error $\text{δ}d_{1-2}$ for an example. Combining Equations (10) and (11), the Δψ error can be expressed by:

(12) $$\Delta {\text{ψ}}_{error}=\Delta \tilde{\psi }-\Delta \text{ψ}=\arctan\left(\frac{d_{1-2}+\text{δ}d_{1-2}}{l_{1-2}}\right)-\arctan\left(\frac{d_{1-2}}{l_{1-2}}\right)$$

**Figure 6:**
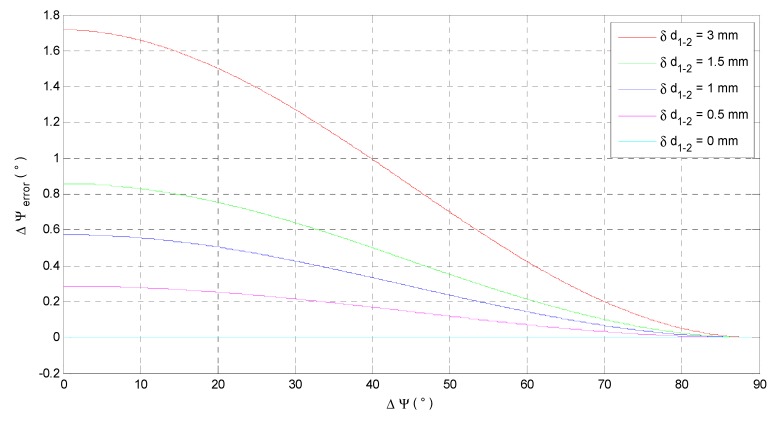
The effect of Δψ on the $\Delta {\text{ψ}}_{error}$.

When $\text{δ}d_{1-2}$ is certain, $\Delta {\text{ψ}}_{error}$ depends on both $l_{1-2}$ and $d_{1-2}$, and $d_{1-2}$ changes with the Δψ. We analyze the effect of $l_{1-2}$ and Δψ on $\Delta {\text{ψ}}_{error}$, respectively, in the cases of the measuring error $\text{δ}d_{1-2}$ = 3 mm, 1.5 mm, 1 mm, 0.5 mm and 0 mm. The distance between LR 1 and 2 $l_{1-2}$ is set as 10 cm. The effects of Δψ on $\Delta {\text{ψ}}_{error}$ are shown in Figure 6.

**Figure 7:**
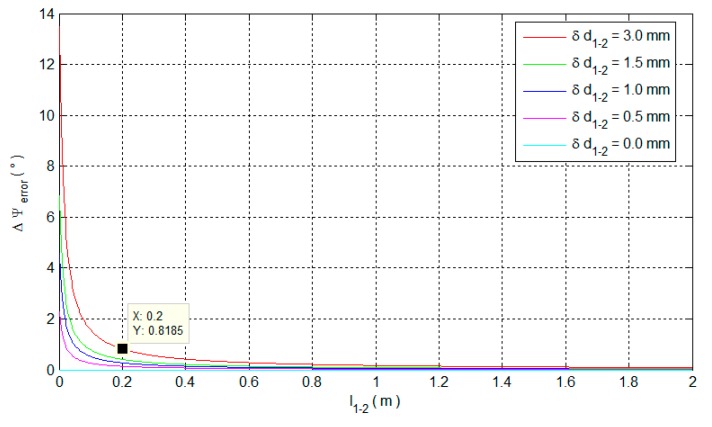
The effect of $l_{1-2}$ on the $\Delta {\text{ψ}}_{error}$.

Figure 6 shows that with a certain measuring error $\text{δ}d_{1-2}$, the $\Delta {\text{ψ}}_{error}$ decreases with the Δψ increasing. Since the $\Delta {\text{ψ}}_{error}$ is related to both Δψ and $l_{1-2}$, the effects of $l_{1-2}$ on $\Delta {\text{ψ}}_{error}$ is maximized when Δψ converges to zero. The influence of $l_{1-2}$ on $\Delta {\text{ψ}}_{error}$ is further investigated when Δψ=0°, as shown in Figure 7.

From the red curve in Figure 7, the $\Delta {\text{ψ}}_{error}$ significantly decreases before $l_{1-2}=0.1$ m and the $\Delta {\text{ψ}}_{error}$ already equals to 0.8185° when $l_{1-2}=0.2$ m. In practice, $\text{δ}d_{1-2}=\text{δ}d_{1}-\text{δ}d_{2}$ can be smaller than 3 mm because $\text{δ}d_{1}$ and $\text{δ}d_{2}$ are random in 1.5 mm~−1.5 mm [8]. Then, the accuracy of the calculated Δψ can be better in the case of $l_{1-2}=0.2$ m, as shown in the curves in other colors.

Notably, the results in the red curve corresponds to the worst situation in both $\text{δ}d_{1-2}$ and Δψ. Hence, the red curve can be a reference to determine $l_{1-2}$ by trading off between the system accuracy and the size. By using the LR aiding system, the steps for determining the LR attitude are:

(1) Find the initial attitude of the three LRs. Put the LR aiding system on a horizontal plane, and then rotate them to emit beams vertical to the wall plane. Observe the measurements from the three LRs while rotate them. When the measurements are equal with acceptable biases, stop rotating the LRs and keep them stationary. We define the attitude of LRs under this circumstance as the initial attitude.

(2) Calculate the initial attitude of the LRs. Because of MEMS gyros noises, only coarse initial alignment is applied. LR aiding system is static on a horizontal plane, so the initial pitch ${\text{θ}}_{0}$ and roll angle $r_{0}$ are assumed to be zero. Then, the initial yaw angle ${\text{ψ}}_{0}$ can be calculated by Equation (9):

(13) $${\text{θ}}_{0}=r_{0}=0,\;\psi_{0}=\arctan\left(\frac{M_{x}^{b}}{M_{y}^{b}}\right)+D$$

(3) Determine the LR 1 attitude. After initial alignment, LR 1 is used to aim at a target by appropriate angular motions. During the angular motions, the pitch and yaw angles of LR 1 can be described as:

(14) $${\text{θ = θ}}_{0}+\Delta \text{θ, ψ}={\text{ψ}}_{0}+\Delta \text{ψ}$$

By substituting Equations (10) and (13) into Equation (14), the LR 1 attitude can be written as:

(15) $$\{\begin{array}{c} \text{θ}=\arctan\left(\frac{d_{1}-d_{3}}{l_{1-3}}\right)\\ \text{ψ}=\arctan\left(\frac{d_{1}-d_{2}}{l_{1-2}}\right)+\arctan\left(\frac{M_{x}^{b}}{M_{y}^{b}}\right)+D \end{array}$$

The LR aiding system works on an assumption that the target is located on a plane wall. However, there may be the instances where the target is on some special structures, such as a column, water pipes, stairs or some decoration with irregular shapes. Besides, when the LRs emit beams on some materials like glass on a wall, LRs can provide no data due to the low reflecting capability of glass. Therefore, a stand-alone LR aiding system is not reliable enough for target positioning. On the other hand, the LR aiding system can provide the attitude with outstanding accuracy. Also, the LR aiding system can provide redundant attitude information in addition to accelerometer/magnetometer aiding system.

## 5. Design of the Federated Kalman Filter

**Figure 8:**
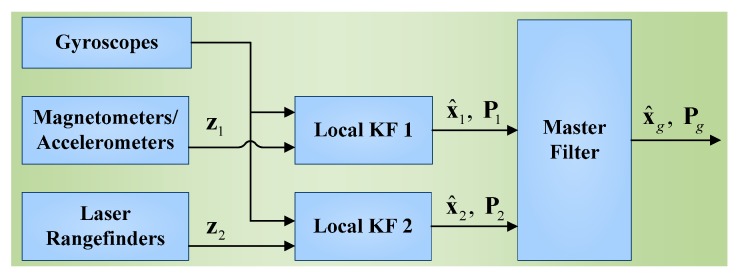
The FKF structure in the target positioning system.

This section involves two designs: (1) local KF models; (2) the FKF configuration. The FKF consists of two Local Filters (LF) and a Master Filter (MF). As a decentralized filter, the FKF can fuse the information that is shared between the LFs and the MF. To implement the system fault-tolerant capability, the no-reset FKF configuration is employed. The structure of the FKF is shown in Figure 8. Two local linear KFs are designed by using the same DCM-based dynamic equations, as well as the observations (*i.e.*, $z_{1}$ and $z_{2}$) from the accelerometer/magnetometer and the LR aiding system, respectively. The two LFs work independently to give estimates (*i.e.*, ${\hat{x}}_{1}$ and ${\hat{x}}_{2}$) and covariance (*i.e.*, $P_{1}$ and $P_{2}$) to the MF. Then, the MF fuses these data to output the globally optimal estimates ${\hat{x}}_{g}$ and $P_{g}$ without feedbacks. The local KFs and the FKF fusion algorithms are presented in details in the following subsections.

### 5.1. Local Kalman Filters

As discussed in Section 4.1, the target positioning accuracy mainly depends on the three functions of the LR attitude angles in Equation (2). In addition, the functions are the second-column elements of the DCM from b-frame to n-frame. Therefore, the DCM-based KF models are designed. Besides, there is no roll-angle motion in the target positioning system, so the DCM from b-frame to n-frame can be simplified as:

(16) $$C_{\text{b}}^{\text{n}}=\left[\begin{array}{ccc} C_{1,1} & C_{1,2} & C_{1,3}\\ C_{2,1} & C_{2,2} & C_{2,3}\\ C_{3,1} & C_{3,2} & C_{3,3} \end{array}\right]=\left[\begin{array}{ccc} \cos\text{ψ} & \cos\text{θ}\sin\text{ψ} & -\sin\text{θ}\sin\text{ψ}\\ -\sin\text{ψ} & \cos\text{θ}\cos\text{ψ} & -\sin\text{θ}\cos\text{ψ}\\ 0 & \sin\text{θ} & \cos\text{θ} \end{array}\right]$$

The differential equation of the DCM can be written as:

(17) $${\dot{C}}_{\text{b}}^{\text{n}}=C_{\text{b}}^{\text{n}}\cdot (\omega_{\text{nb}}^{\text{b}}\times )$$

where:

(18) $$\omega_{\text{nb}}^{\text{b}}=\omega_{\text{ib}}^{\text{b}}-\omega_{\text{in}}^{\text{b}}=\omega_{\text{ib}}^{\text{b}}-C_{\text{n}}^{\text{b}}(\omega_{\text{ie}}^{\text{n}}+\omega_{\text{en}}^{\text{n}})$$

where: 

$\omega_{\text{nb}}^{\text{b}}$ is the angular rate of the b-frame with respect to the n-frame resolved in the b-frame.

$\omega_{\text{ib}}^{\text{b}}$ is the angular rate of the b-frame with respect to the inertial frame resolved in the b-frame, *i.e.*, the measurements from gyros.

$C_{\text{n}}^{\text{b}}$ represents the DCM from the n-frame to the b-frame, *i.e.*, the transpose of $C_{\text{b}}^{\text{n}}$.

$\omega_{\text{ie}}^{\text{n}}$ is the angular rate of the Earth resolved in the n-frame.

$\omega_{\text{en}}^{\text{n}}$ is the angular rate of the n-frame with respect to the e-frame resolved in the n-frame, which depends on the system’s velocity. Since the target positioning system is stationary, this term equals to zero, and then the Equation (18) can be rewritten as:

(19) $$\omega_{\text{nb}}^{\text{b}}=\omega_{\text{ib}}^{\text{b}}-\omega_{\text{in}}^{\text{b}}=\omega_{\text{ib}}^{\text{b}}-C_{\text{n}}^{\text{b}}\omega_{\text{ie}}^{\text{n}}$$

$\left(\omega_{\text{nb}}^{\text{b}}\times \right)$ is the skew symmetric cross-product matrix of $\omega_{\text{nb}}^{\text{b}}$ :

(20) $$(\omega_{\text{nb}}^{\text{b}}\times )=\left[\begin{array}{ccc} 0 & -{\text{ω}}_{z} & {\text{ω}}_{y}\\ {\text{ω}}_{z} & 0 & -{\text{ω}}_{x}\\ -{\text{ω}}_{y} & {\text{ω}}_{x} & 0 \end{array}\right]$$

To simplify the notations, ω is used to represent ${\text{ω}}_{nb}^{b}$ and the subscripts (x, y and z) represent the components of ${\text{ω}}_{nb}^{b}$ resolved in the corresponding axis of the b-frame.

#### 5.1.1. Dynamic Models

The elements of the simplified DCM in Equation (16) are selected as the states of the two LF, which are presented by:

$$x_{i}={\left[\begin{array}{llllllll} C_{1,1} & C_{1,2} & C_{1,3} & C_{2,1} & C_{2,2} & C_{2,3} & C_{3,2} & C_{3,3} \end{array}\right]}^{\text{T}}$$

where i=1, 2 represents LF 1 and 2, respectively. According to Equation (17), the dynamic models are expressed in a matrix form as:

(21) $${\dot{x}}_{i}=Fx_{i}+w_{i}$$

where F is the transition matrix which can be written as:

$$F=\left[\begin{array}{l} \begin{array}{cc} -(\omega_{\text{nb}}^{\text{n}}\times ) & \;\;\;\;\;\;\;0_{3\times 6} \end{array}\\ \begin{array}{ccc} \;\;\;\;0_{3\times 3} & -(\omega_{\text{nb}}^{\text{n}}\times ) & \;0_{3\times 3} \end{array}\\ \;\;\;\;\begin{array}{ccc} 0_{1\times 6} & \;\;\;\;0 & {\;\;\;\;\;\;\;\omega }_{x} \end{array}\\ \begin{array}{ccc} \;\;\;\;0_{1\times 6} & \;\;-{\text{ω}}_{x} & \;\;\;\;\;0 \end{array} \end{array}\right]$$

and $w_{i}$ are the process noise vectors, which are independent Gaussian white noises:

$$w_{i}\sim N(0,Q_{i})$$

where $Q_{i}$ are the process noise covariance matrices.

#### 5.1.2. Observation Models

The accelerometer/magnetometer and the LR aiding systems can provide the pitch and the yaw angles as shown in Equations (9) and (15), which can be used to calculate the elements of DCM as observations. Therefore, the observation models of the LFs can be written as:

(22) $$z_{i}=Hx_{i}+v_{i}$$

where $z_{i}$ represent the observation vectors in the LFs, which are defined as:

$$z_{i}={\left[\begin{array}{cccccccc} \cos{\tilde{\psi }}_{i} & \cos{\tilde{\theta }}_{i}\sin{\tilde{\psi }}_{i} & -\sin{\tilde{\theta }}_{i}\sin{\tilde{\psi }}_{i} & -\sin{\tilde{\psi }}_{i} & \cos{\tilde{\theta }}_{i}\cos{\tilde{\psi }}_{i} & -\sin{\tilde{\theta }}_{i}\cos{\tilde{\psi }}_{i} & \sin{\tilde{\theta }}_{i} & \cos{\tilde{\theta }}_{i} \end{array}\right]}^{\text{T}}$$

where ${\tilde{\theta }}_{1}$ and ${\tilde{\psi }}_{1}$ are the pitch and the yaw angles calculated by using the measurements from the accelerometers and magnetometers, and ${\tilde{\theta }}_{2}$ and ${\tilde{\psi }}_{2}$ are the pitch and the yaw angles calculated by using the measurements from the LRs.

Besides, the observation matrix H in Equation (22) is written as $H=I_{8\times 8}$, and the measurement noise vectors $v_{i}$ are independent Gaussian white noises:

$$v_{i}\sim N(0,R_{i})$$

where $R_{i}$ are the measurement noise covariance matrices.

### 5.2. FKF Fusion

In the no-reset configuration, there are no feedbacks from global estimates to local estimates, as shown in Figure 8. This means that the failures in one of the LFs cannot affect the remaining LF, which allows the system to be highly fault-tolerant. The process of the no-reset FKF fusion can be formulated as follows [34]:

(1) By using upper-bounding approach, the initial local covariance and the local process noise covariance are set to:

(23) $$\begin{array}{l} P_{i,0}={\text{β}}_{i}^{-1}P_{g,0}\\ Q_{i,0}={\text{β}}_{i}^{-1}Q_{g,0} \end{array}$$

where $P_{g,0}$ and $Q_{g,0}$ are the common initial covariance and the process noise covariance, respectively; the fraction values ${\text{β}}_{i}$ sum to unity:

(24) $$\sum_{i=1}^{n}{\text{β}}_{i}=1,{\;\;\;\;\;\;\;0\;<\;\beta }_{i}\leq 1$$

where ${\text{β}}_{1}={\text{β}}_{2}=0.5$.

This approach allows the LF and MF solutions to be statistically independent, so they can be combined to yield the globally optimal solution via relatively simple methods.

(2) The two LFs process their own-sensor measurements via KF algorithms. According to the basic equations of KF [41], the algorithms of the LF are shown as follows:

a. Prediction:

(25) $$\begin{array}{c} {\hat{x}}_{i(k,k-1)}=\Phi_{i(k,k-1)}{\hat{x}}_{i(k-1)}\\ P_{i(k,k-1)}=\Phi_{i(k,k-1)}P_{i(k-1)}\Phi_{i(k,k-1)}^{\text{T}}+Q_{i(k-1)} \end{array}$$

b. Update:

(26) $$\begin{array}{c} K_{i(k)}=P_{i(k,k-1)}H_{i(k)}^{\text{T}}{\left(H_{i(k)}P_{i(k,k-1)}H_{i(k)}^{\text{T}}+R_{i(k)}\right)}^{-1}\\ {\hat{x}}_{i(k)}={\hat{x}}_{i(k,k-1)}+K_{i(k)}(z_{i(k)}-H_{i(k)}{\hat{x}}_{i(k,k-1)})\\ P_{i(k)}=\left[I-K_{i(k)}H_{i(k)}\right]P_{i(k,k-1)} \end{array}$$

where $\Phi_{i(k,k-1)}$ is the discretized state transition matrix, $Q_{i(k-1)}$ is the process noise covariance matrix, $R_{i(k)}$ is the discretized observation covariance matrix, I is the identity matrix, and $K_{i(k)}$ is the filter gain matrix.

(3) The MF fuses the local solutions to provide the globally optimal estimates by using the following equations:

(27) $$\begin{array}{c} {\hat{x}}_{g}=P_{g}\cdot (P_{1}^{-1}{\hat{x}}_{1}+P_{2}^{-1}{\hat{x}}_{2})\\ P_{g}={\left(P_{1}^{-1}+P_{2}^{-1}\right)}^{-1} \end{array}$$

where ${\hat{x}}_{g}$ are the global attitude estimates, which are used to calculate the target position; and $P_{g}$ is the process noise covariance matrix. The no-reset FKF allows the system to be highly fault-tolerant, especially when LF 1 fails due to magnetic disturbances. As a result, the redundant aiding systems and the FKF can allow the system to work robustly, which enhances the practicability and reliability of the system.

## 6. Simulation and Experimental Results

### 6.1. Simulation Results

The positioning accuracy mainly depends on the attitude estimates provided by the local DCM-based KFs. Hence, the performance of the two KFs is presented to show the system accuracy. Then, the system fault-tolerant capability is evaluated by the FKF performance during magnetic disturbances.

#### 6.1.1. Performance of the Two LFs and Positioning Accuracy

**Figure 9:**
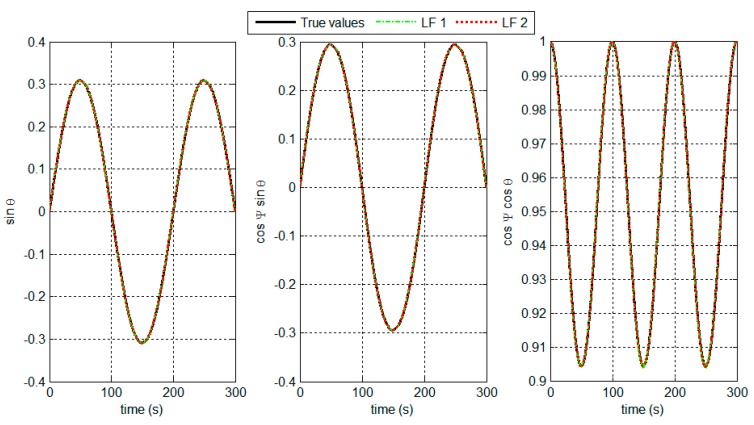
Attitude estimates in the target positioning system.

To test the effectiveness of the DCM-based LFs, the accuracy of the attitude estimates from the two LFs are shown. The main involved conditions are set as follows. The true pitch and yaw angles are set as θ=54°⋅sin(2πt/100) and ψ=54°⋅sin(2πt/100), respectively. The gyros noise ${\text{ε}}_{g}$ is modeled as ${\text{ε}}_{g}={\text{ε}}_{bias}+w_{g}$. The constant bias ${\text{ε}}_{bias}$ is set as 5°/h, and $w_{g}$ is a random white noise of zero mean and 0.5°/h Standard Deviation (SD). The accelerometers noise $\Delta_{a}$ is modeled as $\Delta_{a}=\Delta_{bias}+w_{a}$. The constant bias $\Delta_{bias}$ is set as $10^{-4}g$, and $w_{a}$ is a random white noise of zero mean and $10^{-3}g$ SD. The measuring accuracy of LRs is set as 1.5 mm. Figure 9 shows the results of the attitude estimates, and the Root Mean Square (RMS) error analysis is shown in Table 1.

The results in Figure 9 and Table 1 show that the designed LF 1 and 2 can track suitably the true attitude values. This implies that the target positioning accuracy can be satisfactory.

**Table 1:** RMS error of the attitude estimates.

	LF 1	LF 2
sinθ	0.0010	$7.0733\times 10^{-4}$
cosψsinθ	0.0014	$4.8665\times 10^{-4}$
cosψcosθ	$9.4631\times 10^{-4}$	$7.4538\times 10^{-4}$

**Figure 10:**
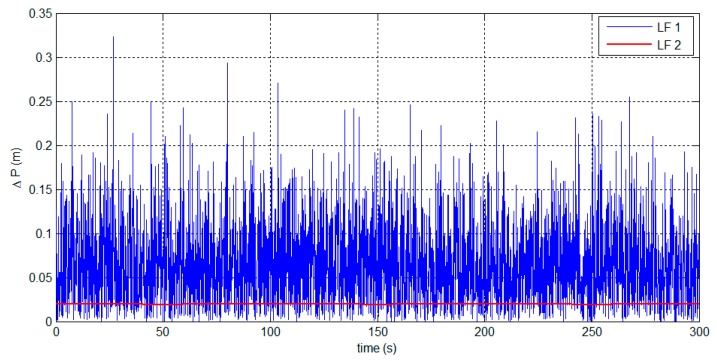
Target positioning errors based on the attitude estimates.

The distance from the observer to the target is set to 80 m. By substituting it into Equation (2), we can obtain the target position. The position error ΔP is used to evaluate the positioning accuracy, which is defined as:

(28) $$\Delta P=\sqrt{{\left(x_{c}-x_{t}\right)}^{2}+{\left(y_{c}-y_{t}\right)}^{2}+{\left(z_{c}-z_{t}\right)}^{2}}$$

where $\left(x_{c},y_{c},z_{c}\right)$ represents the target position calculated by using the attitude estimates, and $\left(x_{t},y_{t},z_{t}\right)$ represents the true target position calculated by using the true attitude. The positioning errors from the attitude estimates are shown in Figure 10 and Table 2.

**Table 2:** Statistical analysis of the positioning errors.

	Mean (m)	SD (m)
**LF 1**	0.06542	0.05014
**LF 2**	0.0195	0.0001697

The LF 2 solutions are smooth and contain less noise. The LF 2 performance benefits from accurate attitude observations from the LR aiding system. By using the attitude estimates, the target positioning accuracy can be less than 0.1 m within 80 m of distance. The simulation results indicate that the LF algorithms can enable the system to perform with high positioning accuracy.

#### 6.1.2. Fault-Tolerant Capability of FKF

As mentioned in Section 4.2.2, the failures of the LR aiding system means that it is unusable. Therefore, fault tolerance refers to the system capability of maintaining effective especially when LF 1 is inaccurate during magnetic disturbances.

**Figure 11:**
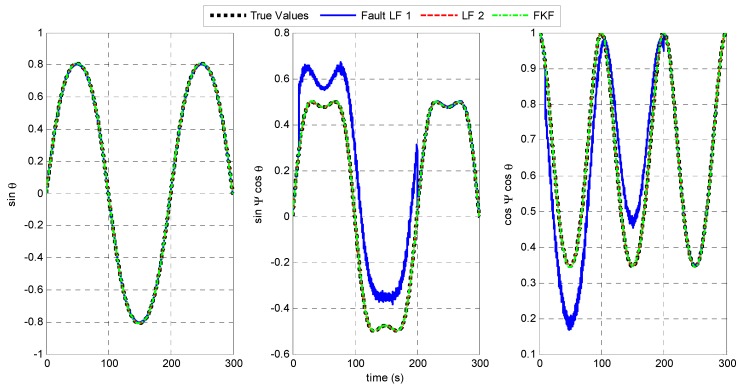
Attitude estimates when LF 1 fails.

Equation (9) shows that magnetic disturbances may result in large errors on the yaw angle. To simulate a failure of LF 1, large errors of yaw angle Δψ=60° with white noise of zero mean and 1° SD is added from 10 to 200 s. The resulting attitude estimates from the fault LF 1, LF 2, and the FKF are shown in Figure 11.

**Figure 12:**
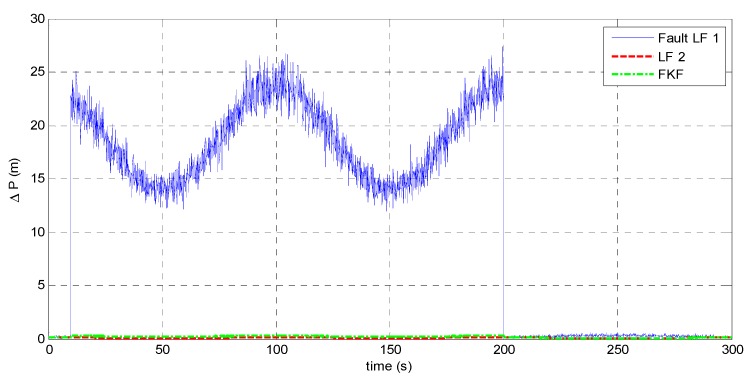
Target positioning errors when LF 1 fails.

Figure 11 shows that during the magnetic disturbances, LF 1 fails to track the true attitude while LF 2 works normally to provide the accurate estimates. Moreover, the FKF fuses the estimates from the two LFs to realize fault-tolerant capability. The target positioning accuracy based on the different filters are shown in Figure 12 and Table 3.

During the magnetic disturbances from 10 to 200 s, the fault LF 1 leads to the significantly large target-positioning errors, namely 18.65 m in average for a target distance of 80 m. However, the FKF makes the system avoid the harmful effect from LF 1. The results show that the positioning error based on FKF is less than 0.3 m.

**Table 3:** Statistical analysis of positioning errors when LF 1 fails.

	Mean (m)	SD (m)
**Fault LF 1**	18.63	3.577
**LF 2**	0.09069	0.006407
**FKF**	0.2573	0.04489

The accuracy performance based on the FKF benefits from: (1) the redundant LF 2 based on the LR aiding system that works normally with high accuracy; (2) the no-reset FKF provides a fault-tolerant way of sharing information from the MARG sensors and the LRs. The results demonstrate the FKF enables the target positioning system to tolerate magnetic disturbances, and then the system robustness is improved.

### 6.2. Experimental Results

Experiments were conducted outside the ÉTS University building where GPS signal visibility is good. The experimental data are logged by using a L3GD20 MEMS gyro, a LSM303DLHC electronic compass which consists of a three-axis accelerometer and a three-axis magnetometer, and a BOSCH GLM80 LR. The measuring accuracy of the LR is ±1.5 mm per 80 m [8], and the MEMS sensors are integrated on a STEVAL-MKI119V1 board. The corresponding datasheets can be seen in [42]. The distance between the observer and the target is 9.42 m.

#### 6.2.1. Accuracy of the Target Positioning System

After initial alignment as illustrated in Section 4.2.2, the LR performs an angular motion to emit a beam to the target on a wall of the building. When positioning the target, stationary data are recorded to obtain the position vector in the n-frame $p_{\text{n}}$ by using Equation (2). A GPS-WAAS receiver can provide the precise position of the observer and the target with regard to the e-frame, which are denoted by $p_{\text{e}}^{\text{o}}$ and $p_{\text{e\_GPS}}^{\text{t}}$, respectively. The target position within e-frame can by calculated by [1]:

(29) $$p_{\text{e}}^{\text{t}}=p_{\text{e}}^{\text{o}}+p_{\text{e}}$$

where $p_{\text{e}}$ can be given by $p_{\text{n}}$, which is detailed in Equation (4). The positioning accuracy can be evaluated by comparing the $p_{\text{e}}^{\text{t}}$ provided by Equation (29) and the measured $p_{\text{e\_GPS}}^{\text{t}}$.

**Figure 13:**
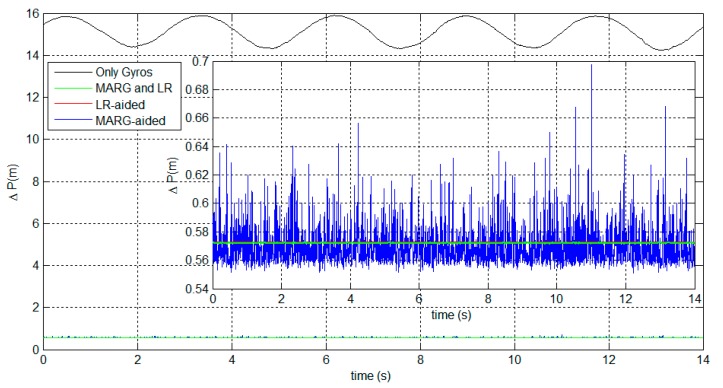
Target positioning errors based on the different strategies.

Figure 13 shows the positioning errors based on different strategies for attitude determination. The strategies refer to: (1) deriving the attitude by only using the gyros (black line); (2) estimating the attitude by using the MARG-based AHRS, *i.e.*, using LF 1 (blue line); (3) estimating the attitude by using the LR-aided AHRS, *i.e.*, using LF 2 (red line); and (4) estimating the attitude by fusing the MARG sensors and LRs, *i.e.*, using the FKF (green line). Table 4 shows the statistical analysis of the positioning errors.

The results show that both the attitude estimates based on the two aiding systems significantly improve the positioning accuracy. It firstly implies that the designed DCM-based KF can provide accurate attitudes for target positioning. Secondly, the proposed LR aiding system is effective and useful in practical applications due to its remarkable accuracy.

Moreover, the FKF-based positioning system can effectively reduce by about 93.3% the positioning error compared to the stand-alone gyros system. Also, the FKF-based system exhibits more stable accuracy than the MARG-based AHRS, which benefits from the LR aiding system with little noise. These indicate that the FKF algorithms can appropriately fuse the two LFs in terms of the final positioning accuracy.

**Table 4:** Statistical analysis of positioning errors.

	Mean (m)	SD (m)
**Only Gyros**	15.12	0.5471
**MARG-aided**	0.5698	0.01404
**LR-aided**	0.5726	$8.098\times 10^{-11}$
**MARG and LR**	0.5721	0.000169

The results show that the proposed system based on FKF can significantly improve the positioning accuracy. The positioning error is less than 0.6 m for a 9.42 m distance fixed between the observer and the target. However, there is a gap between the simulation and the experimental results. From Table 2, the theoretical accuracy for a distance of 80 m is already nine times higher than the experimental accuracy for a distance of 9.42 m. There are two reasons accounting for this gap. Firstly, only a coarse initial alignment is applied in the proposed system. The initial attitude errors are then propagated through the system algorithm and thus cause large positioning errors. However, high-accuracy initial alignment based on MEMS sensors is a challenge due to the large inherent sensor noises. A large amount of research has been conducted to improve the accuracy of MEMS-based initial alignment. Therefore, to further improve the positioning accuracy, the initial alignment based on MEMS sensors will be studied as a separate topic in the future. Secondly, the laser beam of LR 1 is assumed to align with the *Y_b_* axis of the b-frame in the system. The errors of the installation angle will break the laws of the target positioning algorithm in Equation (2) and then the positioning accuracy is reduced. Therefore, the installation errors cannot be ignored and some effective calibration approaches like those described in [43] should be added to the proposed system.

#### 6.2.2. System Fault-Tolerant Capability

**Figure 14:**
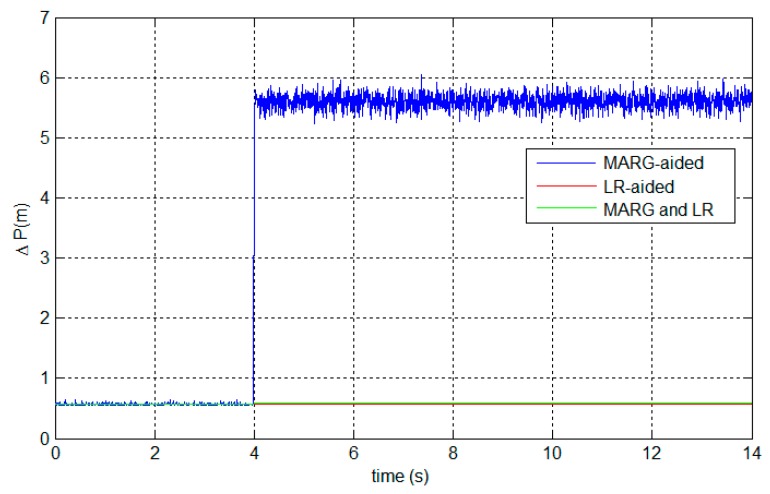
Fault-tolerant capability of the proposed system.

Simulated magnetic disturbances are added to the experimental data from 4 s to the end. The simulated disturbances are set as 0.5 G with a white noise of zero mean and 10^−2^ G SD. Figure 14 presents the positioning errors to show the fault tolerance. Table 5 gives the statistical analysis of the positioning errors during the disturbances.

**Table 5:** Statistical analysis of positioning errors during the magnetic disturbances.

	Mean (m)	SD (m)
**MARG-aided**	5.609	0.1231
**LR-aided**	0.5726	$8.098\times 10^{-11}$
**MARG and LR**	0.5901	0.000271

It can be seen from Figure 14 and Table 5 that, MARG-based AHRS causes large positioning errors during magnetic disturbances, which complies with the theoretical analysis in Section 6.1. However, the fault-tolerant FKF can protect the system from the magnetic disturbances. Also, the FKF-based system accuracy remains quite satisfactory.

The fault-tolerant accuracy performance results from the no-reset configuration of the FKF, as well as the high accuracy of the LR aiding system as shown in Figure 14 and Table 5. The LR-aided AHRS possesses high accuracy, while the MARG-based AHRS can work almost everywhere without laser limitations, even in some situations where the LR aiding system fails. The FKF-based system can fuse the two complementary attitude aiding systems. The FKF fusion algorithm enables our system to benefit from the advantages of both aiding systems, as well as avoid the faults caused by a single aiding system. The system robustness is demonstrated by the fault-tolerant results obtained during the magnetic disturbances.

## 7. Conclusions

This paper has presented a target positioning system based on MEMS MARG sensors and LRs. Two main issues have been addressed to improve the system performance: target-positioning accuracy and fault-tolerant capability. The linear DCM-based KF algorithms are designed to limit the errors of the LR’s attitude. Then, the accelerometer/magnetometer and the LR attitude aiding system are introduced. The LR aiding system is especially designed based on three LRs, which can accurately determine attitude. Two local DCM-based KFs can be designed based on the two aiding systems, respectively. Then, the no-reset FKF is used to fuse the redundant attitude estimates from the two independent local KFs. The FKF fusion algorithms enable the system to work effectively even when one of the LFs has faults. The simulation and experimental results demonstrate that the proposed system can improve target-positioning accuracy and perform with high robustness.

The designed DCM-based KF could be applied in target positioning systems based on distance/attitude measurements. Additionally, the LR aiding system could be exploited in the situation where the plane construction is available, for instance in indoor navigation and localization. Future work should benefit greatly by developing accurate MEMS-based initial alignment and the calibration for the installation errors.
